# Case Report: Exploration of changes in serum immunoinflammation-related protein complexes of patients with metastatic breast cancer

**DOI:** 10.3389/fonc.2023.1207991

**Published:** 2023-07-21

**Authors:** Chang Chen, Yali Xu, Zhizhen Lai, Zhili Li, Qiang Sun

**Affiliations:** ^1^ Department of Breast Surgery, Peking Union Medical College Hospital, Chinese Academy of Medical Sciences and Peking Union Medical College, Beijing, China; ^2^ Department of Biophysics and Structural Biology, Institute of Basic Medical Sciences, Chinese Academy of Medical Sciences and School of Basic Medicine, Peking Union Medical College, Beijing, China

**Keywords:** immunoinflammation-related protein complexes, breast cancer, serum, biomarker, disease surveillance

## Abstract

Patients with advanced breast cancer are difficult to treat and have poor prognosis. At present, the commonly used methods to monitor the disease progression of breast cancer are imaging examinations such as breast ultrasound, mammography and peripheral blood tumor markers such as carcinoembryonic antigen (CEA) and carbohydrate antigen 15-3 (CA15-3). However, none of them can detect tumor progression at an early stage. Serum immunoinflammation-related protein complexes (IIRPCs) showed potential to indicate cancer progression. Therefore, we attempted to monitor the level of IIRPCs in peripheral blood of patients with metastatic breast cancer and compare it with patients’ treatment and disease progression, and here we performed case reports of two of them.

## Introduction

In recent years, the breast cancer has become the second leading cause of cancer death among women after lung cancer in the United States (1). It is also the most common cancer and the sixth leading cause of cancer death in Chinese women (2). Recurrent and metastatic breast cancer is currently the most difficult part to diagnosis and treatment. The sensitivity of imaging tests (such as ultrasound, mammography, MRI, and PET/CT) and peripheral blood tumor markers currently used in clinical practice is not high enough (3–5). Early detection of disease progression is also a hot topic of current research. Blood components such as free DNA and RNA, proteins, peptides, circulating tumor cells, and metabolites, are indicators of health status as well as disease status (6). And protein complexes assembled by non-covalent bond interactions have already showed potentials in differentiation of disease in different status (7–9). Disease-specific immune response-related protein complexes in the blood are associated with disease conditions, especially for cancer (10). Previous study has revealed that inflammation regulated by immunoglobulin and immune complexes might be a functionally significant factor in cancer progression (11). Serum immunoinflammation-related protein complexes (IIRPCs) were found significantly different between cancer patients and healthy control, which including immune-related proteins, inflammation-related proteins, and complement-related proteins (12). The levels of IIRPCs in non-small cell lung cancer patients had been found to vary with cancer progression (13). And monitoring IIRPCs may help guide therapeutic management (13). We therefore attempted to monitor IIRPCs in patients with metastatic breast cancer to explore whether it could reflect disease changes.

## Case description

### Case 1

**Figure 1:**
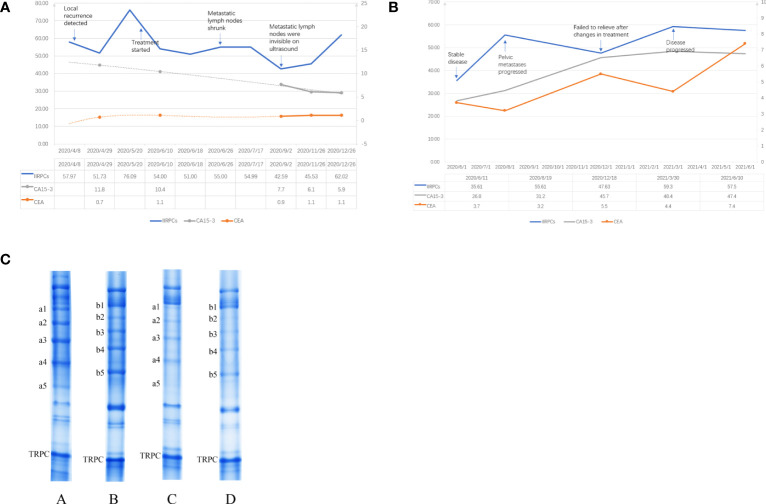
**(A)** Change trends in the levels of the IIRPCs, CEA, and CA15-3 during follow-up of case 1. The left vertical coordinate applied to the values of IIRPCs, the right vertical coordinate applied to the values of CEA (ng/ml) and CA15-3 (U/ml). **(B)** Change trends in the levels of the IIRPCs, CEA, and CA15-3 during follow-up of case 2. The left vertical coordinate applied to the values of IIRPCs and CA15-3 (U/ml), the right vertical coordinate applied to the values of CEA (ng/ml). **(C)** Examples of serum IIRPCs with or without tumor metastasis. The amount of serum IIRPCs relative to serum TRPC in each gel was quantified. The gray value of IIRPCs in metastasis patients (A and B) is substantially higher than in disease-free patients (C and D).

The first case was a 54-year-old woman who underwent a modified radical mastectomy for left breast cancer in May 2018 with a postoperative pathology of invasive ductal carcinoma (IDC). The maximum diameter of her tumor was 4 cm and the axillary lymph node had metastasis (2/18). Immunohistochemical results were ER negative, PR negative, HER-2 positive and ki67 40%. Postoperatively, the patient did not undergo further radiotherapy, chemotherapy or targeted therapy for personal reasons. The patient had no significant abnormalities on postoperative review until January 2020, when the imaging revealed left chest wall recurrence and multiple lymph node metastases in the left supraclavicular area, left axilla, and left internal mammary artery. She came to our hospital in April of the same year. At the time of the patient’s visit, we retained a specimen of her peripheral blood for monitoring of IIRPCs, and blood samples were retained at each subsequent visit for blood testing. Serum protein complexes were isolated using native polyacrylamide gel electrophoresis (PAGE), which was described in detail in our previous article (12). The quality control (QC) sample was a mixture of serum from 5 random patients. An UMAX PowerLook 2100XL scanner (Techville, Inc., USA) was used to scan the stained gel for quantification based on optical density. The gray value of each band was calculated using Quantity One software (version 4.6.3, Bio-Rad). The levels of bands in each gel were exported into Microsoft Excel after the gel background had been subtracted. Previous research had shown that the transferrin-related protein complex (TRPC) had no statistical differences in patients with different sex, age, patterns of the IIRPCs, or health status (12), so the TRPC can be as an internal reference for quantifying the IIRPCs. We quantified the gray value of TRPC in each sample relative to the TRPC of the QC sample to ensure consistency, and then normalized it to 100. After that the amounts of serum IIRPCs relative to serum TRPC in each gel were quantified and calculated. We recommended chemotherapy in combination with targeted therapy to her. And she began treatment in June 2020 after careful consideration. **Figure 1A** demonstrates the changes in IIRPCs levels at each monitoring visit and the changes in peripheral blood tumor markers over the corresponding time period. The level of IIRPCs rose to peak before treatment and then dramatically fell. The patient’s condition significantly relieved after treatment. Subsequent ultrasound review revealed that the metastatic lymph nodes were significantly reduced and almost invisible. Both carcinoembryonic antigen (CEA) and carbohydrate antigen 15-3 (CA15-3) were at normal levels during the same period and did not change with disease.

### Case 2

The second case was a 48-year-old woman who underwent a modified radical mastectomy for right breast cancer in 2014. The postoperative pathology was invasive micropapillary carcinoma (5*3.5*2.5cm) with axillary lymph node metastasis (7/36). Immunohistochemistry results were ER negative, PR negative, HER2 positive and ki67 40%. The patient underwent 6 courses adjuvant chemotherapy (docetaxel + cyclophosphamide + pirarubicin) and one year targeted (Herceptin) therapy after surgery. Chemotherapy was followed by adjuvant radiotherapy. By 2018, the patient’s review revealed chest wall recurrence, multiple lymph node metastases (bilateral supraclavicular, bilateral axillary, neck), and pericardial metastasis. After therapy with docetaxel + pyrotinib, the patient’s condition stabilized. At her visit to our hospital in June 2020 we retained her peripheral blood sample for IIRPCs testing and dynamic monitoring. However, the patient’s pelvic metastases progressed two months later and failed to relieve even after changes in treatment regimen. The detection method for IIRPCs and the quantification method were the same as in case 1. **Figure 1B** demonstrated the dynamic changes of IIRPCs as well as tumor markers of this patient. The quantitative detection of IIRPCs found its level was elevated in response to disease progression. The patient’s condition alleviated unsatisfactorily in the subsequent treatment and the level of IIRPCs did not decrease to the baseline(the level at the first sampling, shown in **Figure 1B**). In this case, her CEA and CA15-3 levels also increased significantly with disease progression.

## Discussion

Serum IIRPCs protein complexes are composed of variable inflammation and immunity related protein molecules through non-covalent interactions, mainly including complement factor H, complement C3, C4, C7 components, haptoglobin, immunoglobulin α, γ, and κ components, apolipoprotein A-I and so on (12). It has been shown that peripheral blood complement and immunoglobulins are associated with the development and progression of cancer (10, 14–16). And previous studies have found significant differences in the levels of IIRPCs between tumor patients and healthy individuals (12). In patients with advanced non-small cell lung cancer, changes in the levels of IIRPCs were associated with patient response to treatment and changes in disease status (13). We selected some breast cancer patients to initially explore whether the IIRPCs levels and patterns were associated with breast cancer disease progression.

The results of our pre-experiment found that the IIRPCs bands patterns of all breast cancer patients were classified into seven major patterns (from a to g), which were consistent with previous studies (12, 13, 17). And no new banding pattern was identified. The patients in our cases had no severe infection, other primary tumor, pregnancy, lactation, other serious medical conditions, or other diseases known to be significantly associated with IIRPCs. The patterns of IIRPCs in our assay did not correlate significantly with patient age, tumor stage, grade, and whether metastasis was present. Therefore we mainly want to monitor the changes in the levels of IIRPCs dynamically. We initially found higher concentrations of IIRPCs protein bands in patients with metastatic breast cancer than in patients without metastasis (as shown in **Figure 1C**). As mentioned above, IIRPCs contains many immune-inflammatory related proteins including haptoglobin, complements. It has been found that higher level of haptoglobin was related to pooler survival of breast cancer (18). And haptoglobin could regulated cell cycle progression and apoptosis in breast cancer cells (19). Lisa J had reported the level of apolipoprotein A-I might be positively correlated with the risk of breast cancer (20). Complement C3 also had been researched as a predictive biomarker in breast cancer (21).

In the first patient, IIRPCs levels decreased after treatment, consistent with the patient’s remission. In contrast, the second patient responded poorly to treatment, failed to achieve disease remission, and IIRPCs remained at high levels. However, tumor markers represented by CEA and CA15-3 were consistently elevated with disease progression only in the second patient. We think it may be because the second patient had more metastatic lesions and the first patient had only local recurrence and regional lymph node metastasis. The sensitivity of CEA and CA15-3 for detecting disease progression in breast cancer is not high (22, 23). So personalized IIRPCs could be a potential indicator that reflects the tumor burden in breast cancer patients, which might have good sensitivity. We will continue to collect samples for IIRPCs assays and will follow up with statistical analysis to further explore the role of IIRPCs

## Data availability statement

The raw data supporting the conclusions of this article will be made available by the authors, without undue reservation.

## Ethics statement

Written informed consent was obtained from the individual(s) for the publication of any potentially identifiable images or data included in this article.

## Author contributions

CC performed the result analysis and wrote the draft manuscript. YX and ZLL designed the study. CC and ZZL performed the experiments and the data analysis. QS edited the draft. All authors contributed to the article and approved the submitted version.
